# Auditory perception in the aging brain: the role of inhibition and facilitation in early processing

**DOI:** 10.1016/j.neurobiolaging.2016.06.022

**Published:** 2016-11

**Authors:** George Stothart, Nina Kazanina

**Affiliations:** School of Experimental Psychology, University of Bristol, Bristol, UK

**Keywords:** Aging, Auditory event-related potentials, Inhibition, Speech, Audio-visual, N2, P3a

## Abstract

Aging affects the interplay between peripheral and cortical auditory processing. Previous studies have demonstrated that older adults are less able to regulate afferent sensory information and are more sensitive to distracting information. Using auditory event-related potentials we investigated the role of cortical inhibition on auditory and audiovisual processing in younger and older adults. Across puretone, auditory and audiovisual speech paradigms older adults showed a consistent pattern of inhibitory deficits, manifested as increased P50 and/or N1 amplitudes and an absent or significantly reduced N2. Older adults were still able to use congruent visual articulatory information to aid auditory processing but appeared to require greater neural effort to resolve conflicts generated by incongruent visual information. In combination, the results provide support for the Inhibitory Deficit Hypothesis of aging. They extend previous findings into the audiovisual domain and highlight older adults' ability to benefit from congruent visual information during speech processing.

## 1 Introduction

Aging affects auditory perception in a diverse and multi-faceted manner. Presbycusis is a general term that refers to high-frequency age-related hearing loss and is present in approximately 50% of adults aged over 70 (Roth et al., 2011). It is typically characterized by a progressive loss of hearing that begins in the high-frequency ranges and subsequently advances into the middle and lower frequencies (Gates and Mills, 2005). Interestingly, auditory ability as measured via puretone-hearing threshold levels (HTLs) does not straightforwardly correlate with functional performance by older adults on auditory tasks (Schneider and Pichora-Fuller, 2000, Tun et al., 2012). Instead, functional deficits in older adults, such as impaired frequency discrimination (Schneider and Pichora-Fuller, 2000), gap detection (Schneider et al., 1994, Schneider et al., 1998), and greater sensitivity to noise during speech perception (Helfer and Freyman, 2008, Tun et al., 2012) are better predicted by measures of executive function (Akeroyd, 2008, Houtgast and Festen, 2008, Humes, 2005). That is, frontal cortical areas appear to compensate for reduced peripheral auditory and auditory cortex activity (Wong et al., 2009), although the frontal lobe itself shows the greatest age-related linear degeneration in the cortex (Raz and Rodrigue, 2006, Raz et al., 2005). The emerging picture is that auditory perception and cognition involve a complex interplay between peripheral and central systems, each undergoing age-related changes (see also Anderson et al., 2013, Bidelman et al., 2014).

The Inhibitory Deficit Hypothesis (IDH) of cognitive aging proposes that age-related deficits in performance across a wide range of perceptual, attentional, and cognitive tasks stem from an inability to inhibit the processing of irrelevant information (Hasher and Zacks, 1988). Three functions of inhibition have been distinguished: (1) controlling access of irrelevant information to the focus of attention and working memory, (2) deleting irrelevant information from attention and working memory, and (3) suppressing or restraining strong but inappropriate responses (Guerreiro et al., 2010, Hasher et al., 2007). The second and third aspects of inhibitory deficit have been well demonstrated in many studies in which older adults are able to facilitate and enhance the processing of relevant visual information, yet are unable to efficiently ignore irrelevant information (e.g., Gazzaley et al., 2005, Gazzaley et al., 2008, Vallesi et al., 2009). In addition, these deficits are to some extent reversible with training that boosts frontal lobe activity (Anguera et al., 2013). Less well-established, particularly in the auditory modality, are the effects of age on the first subcomponent of inhibition, that is, controlling access to the focus of attention, We used the P50, N1, P2, N2, mismatch negativity (MMN) and P3a event-related potentials (ERPs) to examine the role of inhibition in controlling access to the focus of attention in early perceptual processing in young and older adults. The earliest component, P50, typically peaks between 40 and 70 ms, is generated bilaterally in the primary auditory cortex (Liegeois-Chauvel et al., 1994, Reite et al., 1988, Weisser et al., 2001) and reflects the regulation of sensory information from the peripheral nervous system to the cortex. The N1 typically peaks between 90 and 130 ms, and like the P50, is generated bilaterally in the primary and association auditory cortex, with generators in the transverse temporal gyri (Picton et al., 1999, Scherg et al., 1989). The P2 peaks between 150 and 200 ms with generators in the auditory association cortex and is responsive to complex acoustic features, and its modulation by learning and expertise. (Pantev et al., 1988, Shahin et al., 2005). The P50-N1-P2 complex reflects the information flow from primary auditory to association cortical processing, and the transition from tonotopic auditory processing to more complex spectral processing, and greater sensitivity to top-down regulation. The N2 family comprises the standard N2, the N2a, and the N2b. These subcomponents appear between 200 and 350 ms, and their topography, neural sources, and proposed function varies depending on subtype (Folstein and Van Petten, 2008). We focussed on the standard N2, typically observed in response to stimuli that involves inhibitory processing, for example, ignoring the standard stimulus in an oddball task (Bertoli and Probst, 2005). It has a fronto-central distribution with neural sources in the right orbito-frontal cortex and anterior cingulate (Falkenstein, 2006, Näätänen and Picton, 1986). Deficits in inhibitory processing due to age or pathology (e.g., depression, alcoholism) have been associated with reduced or absent standard N2 (Bertoli and Probst, 2005, Kaiser et al., 2003, Pandey et al., 2012, Wascher et al., 2011). The MMN represents the detection of change, in particular in the sensory environment (Escera and Coral, 2007), and can initiate the orientation of attention to novel or unexpected stimuli (Näätänen and Michie, 1979, Näätänen et al., 1978). It is calculated by subtracting the response to the frequently presented standard stimulus from that of a rare deviant stimulus and is typically characterized by a negative deflection in this difference wave during the period of 100–250 ms with neural sources located bilaterally in the superior temporal gyri and frontal lobes (for a review see Deouell, 2007). Finally, the P3a component provides the first index of selective attentional orientation and is proposed to represent the updating of working memory representations of incoming stimuli. The P3a has a fronto-central topography and is elicited in response to task-irrelevant rare events (Katayama and Polich, 1998).

Age affects these ERPs differentially. Early P50 and N1 amplitudes have been shown to increase and latencies decrease in older adults, explained as a reduction in frontal regulation of afferent sensory input (see Friedman, 2008 for a comprehensive review). No demonstrable pattern emerges for P2, while there is limited but consistent evidence for a reduction in N2 amplitudes (Ceponiene et al., 2008). Oddball paradigms are valuable tools in assessing both distraction and inhibition in older adults. Measures of distraction, for example, P3a or incorrect behavioral responses to task-irrelevant deviant stimuli, have been demonstrated to be slower in older adults (Andrés et al., 2006). Inhibition can be observed in oddball paradigms through responses to repeating, task-irrelevant standard stimuli. In aging, the P50 or N1 amplitudes to standard stimuli have been shown to be enhanced as a result of less efficient inhibition of irrelevant information (Friedman, 2008), whereas the N2 response to standard stimuli which is considered to reflect a halt in the processing of an irrelevant stimulus (see Section 4) has been shown to be reduced or absent in older adults (Bertoli and Probst, 2005). There is mixed evidence for age-related changes to MMN, with considerable variance in experimental design, analysis techniques and controlling for age-related hearing loss contributing to conflicting results (see Cheng et al., 2013 for a meta-analysis and review). The P3a response is typically reduced or delayed in healthy aging, suggesting a reduced attentional orientation response in older adults (Czigler et al., 2006, Fabiani and Friedman, 1995; see Friedman, 2008 for a review; Knight, 1987, Walhovd and Fjell, 2001). However, there is evidence that the P3a habituates in younger adults, but not in older adults, suggesting that attentional capture by rare or novel stimuli may be greater in aging (Alperin et al., 2014, Friedman et al., 1998).

Speech processing provides a useful tool to examine whether the effects of age on perception and attention in auditory processing extend to audiovisual processing and whether older adults are still able to benefit from the presence of visual cues and “facilitate” the processing of relevant sensory information. As an experimental stimulus, speech is equally ecologically valid in both its audiovisual and auditory forms. Viewing a speaker while listening to continuous speech has been shown to be equivalent to a 15 dB increase in the auditory signal (Sumby and Pollack, 1954), whereas simply observing silent visual speech articulation activates the primary and association auditory cortices (Calvert et al., 1997). The boost given to auditory processing by visual information is however dependent on the congruency and predictive value of the visual articulation (van Wassenhove et al., 2007, Winneke and Phillips, 2011). It appears that much of the benefit derived from multisensory speech is maintained in aging. Older adults' behavioral performance in speech perception studies using multisensory and unisensory speech stimuli has been demonstrated to be comparable to younger adults (Sommers et al., 2005, Tye-Murray et al., 2010) and their sensitivity to the McGurk illusion as equivalent to younger adults (Cienkowski and Carney, 2002, Huyse et al., 2014). It has been proposed that audiovisual integration is exceptionally robust to, and may even be enhanced by, aging, and that enhancement may be a compensatory process for unisensory processing deficits (Diederich et al., 2008, Peiffer et al., 2007, Winneke and Phillips, 2011). What remains to be addressed is how multisensory processing performance in older adults contributes to more general theories of cognitive aging. Following the predictions of the IDH, congruent visual information should aid auditory processing and be maintained in older adults, and incongruent visual information however should serve as a greater distractor to older adults.

We conducted 2 experiments to investigate the role of facilitation and inhibition in auditory processing in aging. Experiment 1 examined the effects of age on auditory processing of puretone and natural speech stimuli. We hypothesized that older adults experience deficits in the inhibition of auditory information that would manifest as increased early sensory responses (P50 and N1), as a consequence of reduced frontal lobe regulation of afferent sensory information. In addition, we hypothesized that older adults would show a reduced N2 to standard stimuli and reduced auditory mismatch negativity (aMMN) as a consequence of their inability to successfully ignore or inhibit the processing of repeating standard stimuli. Experiment 2 examined whether the patterns of age-related change observed in experiment 1 extended to audiovisual speech processing. In addition, by manipulating the congruency of the accompanying visual information, we were able to examine the influence of “relevant” versus “distracting” visual information. We hypothesized that older adults should still be able to facilitate relevant information, that is, congruent visual information but would show greater distraction or interference from incongruent visual information.

## 2 Experiment 1

The experiment consisted of 2 paradigms. First, puretones were presented in a passive listening paradigm providing measures of basic auditory processing (P50, N1, and P2) and inhibitory processing (N2). Second, natural speech syllables were presented in an oddball paradigm which in addition to the measures provided by the passive listening paradigm (P50, N1, P2, and N2) also provided measures of change detection and attentional orientation (MMN and P3a, respectively, to task-irrelevant deviant stimuli).

### 2.1 Participants

Twenty younger adults (aged 18–23, mean age 19.5 [±1.5], 5 males) and 26 healthy older adults (aged 62–88, mean age 76.0 [±7.0], 14 males) gave consent to participate in the study. Younger adults were recruited from the University of Bristol student population and declared themselves to be in normal health. Older adults were recruited by the Avon and Wiltshire and South Gloucestershire Primary Care Trust memory service clinics at the Bristol Research into Alzheimer's and Care of the Elderly Centre, Frenchay Hospital, and the Research Institute for the Care of Elderly People, Royal United Hospital, Bath. They participated as part of a wider study into dementia as healthy controls. Each older adult was assessed by memory clinic staff and displayed normal cognitive function in relation to their age and educational attainment (mean mini-mental state examination Score = 28.5/30 [±1.2]) and none met clinical criteria for dementia or any other neuropsychological disorder. No older adults had history or signs of stroke or transient ischemic attack, significant head injury, depression, or other psychiatric disorder, or major neurological disease, and none were receiving medication (prescribed or non-prescribed) deemed likely to affect cognitive function. All had normal or corrected-to-normal vision and were right hand dominant. All appropriate approvals for our procedures were obtained from the National Research Ethics Service Committee South West-Bristol, Ref. 09/H0106/90. Participants provided written informed consent before participating and were free to withdraw at any time.

### 2.2 Stimuli

#### 2.2.1 Puretones

Stimuli were 1000-Hz puretones presented binaurally through headphones at a fixed volume of approximately 60-dB sound pressure level (SPL). The duration of the tones was 200 ms with a mean interstimulus interval (ISI) of 560 ms, varying randomly between 480 and 640 ms.

#### 2.2.2 Auditory speech

Stimuli were digitally recorded samples (audio sample rate: 44.1 KHz in 16 bits) of a female speaker pronouncing the syllables /ba/ (standard), /da/ (deviant), and /bi/ (target). The /ba/ syllable was the audio recording taken from the /ba/ video used in the audiovisual paradigm (see experiment 2) ensuring that the standard stimuli were acoustically identical in both experiments. Stimuli were presented binaurally through headphones at approximately 60-dB SPL above the participant's HTL. The duration of the stimuli was 325 ms with a mean ISI interval of 620 ms, varying randomly between 520 and 720 ms. Stimuli were matched for intensity using Praat software (Boersma and Weenink, 2009).

### 2.3 Procedure

#### 2.3.1 Hearing threshold level assessment and adjustment

Participants' HTLs were assessed using a Bekesy threshold procedure (Haupt, 2003). The auditory standard /ba/ and deviant /da/ stimuli were used as stimuli in the HTL test rather than puretones to provide an ecologically appropriate measure of HTL. Puretone stimuli were presented at a fixed 60-db SPL and not adjusted for individuals' HTL. Auditory speech stimuli were presented at approximately 60-db SPL above the participant's individual HTL, which required an increase in the stimuli SPL by 8.98 (±2.82) dB for younger adults and 17.80 (±7.33) dB for older adults. This ensured that any age-related differences observed in responses to the auditory-only or audio-visual speech could be compared against a paradigm in which HTL had not been adjusted to dissociate the effects of age from the effects of the physical intensity of the stimulus. In addition, correlational analyses between ERP amplitudes and HTL are presented in Supplementary Material.

##### 2.3.1.1 Puretones

Participants were instructed to listen to the tones, to not respond in any way, and to maintain their gaze at a fixation point on the monitor. Two hundred tones were presented.

##### 2.3.1.2 Auditory speech

Participants were instructed to maintain their gaze at a fixation cross in the centre of the screen while listening to a continuous stream of syllables, consisting of the frequent standard syllable /ba/ interspersed with an infrequent deviant /da/ and infrequent target /bi/. They were asked to press a button in response to the target stimulus. They were instructed to ignore the standard and deviant stimuli. The target and deviant stimuli were presented in a pseudo-random sequence among the standards with at least 2 standards preceding each deviant. Eight hundred and ninety six standards, 112 deviants (i.e., standard:deviant ratio = 8:1) and 8 targets were presented in 2 blocks lasting 8 minutes each. (Initially, no target stimulus was included to exactly match the audiovisual paradigm in experiment 2. However, pilot data revealed that the lack of task, combined with the lack of visual stimulation led to participants becoming drowsy and subsequent overwhelming alpha wave contamination of the evoked potentials. Therefore, a rare target stimulus was introduced to maintain the attentional and physiological arousal of the participant. The number of target stimuli was very low to maintain as much congruity with the audiovisual paradigm as was possible.)

#### 2.3.2 EEG recording

Electroencephalographic (EEG) signals were sampled at 1000 Hz from 64 Ag/AgCl electrodes fitted on a standard electrode layout elasticized cap using a BrainAmp DC amplifier (Brain Products GmbH) with a common FCz reference and online low-pass filtered at 250 Hz. Impedances were below 5kΩ. Recordings were analyzed offline using Brain Electrical Source Analysis software v5.3 (BESA GmbH). Artifacts including blinks and eye movements were corrected using BESA automatic artifact correction (Berg and Scherg, 1994), and any remaining epochs containing artifacts ≥100 μV were rejected. The rejection rate never exceeded 10% of trials for each participant and stimulus.

#### 2.3.3 EEG analysis

**Fig. 1:**
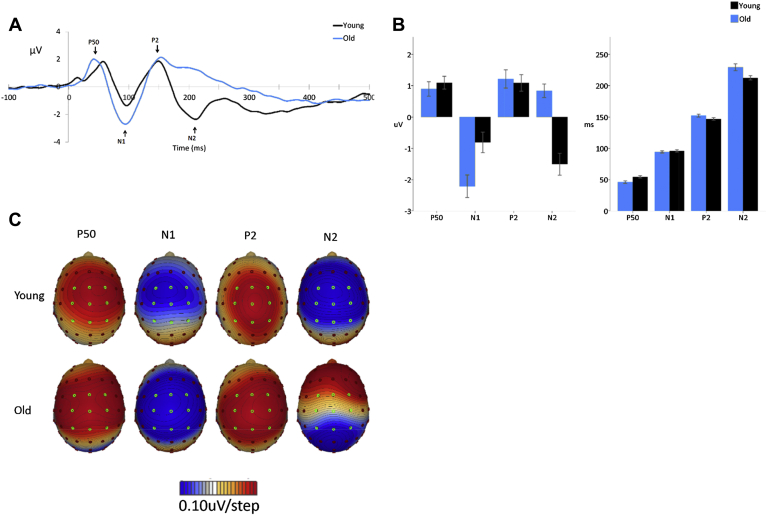
(A) Grand average responses to puretone stimuli measured at the vertex region of interest (average of electrodes FC1, FCz, FC2, C1, Cz, C2, CP1, CPz, and CP2) for younger and older adults, experiment 1. (B) Mean amplitudes and latencies of the ERPs elicited in response to puretones, for younger and older adults. Error bars indicate the standard error of the mean. (C) Topological plots of the ERPs to puretones for younger and older adults. Electrodes included in the vertex region of interest are highlighted in green. Abbreviation: ERPs, event-related potentials. (For interpretation of the references to color in this figure legend, the reader is referred to the Web version of this article.)

Data were re-referenced offline to a virtual linked mastoid reference, using BESA spherical spline interpolation (BESA GmbH). Epochs from −100 to 500 ms around stimulus onset were defined for the auditory and puretone data. Given the well-established scalp distribution of auditory ERPs (i.e., peak amplitude typically occurring at the vertex) and after confirmation via examination of the topography of each component in each group (see Fig. 1C), the values of 9 electrodes (FC1, FCz, FC2, C1, Cz, C2, CP1, CPz, and CP2) were averaged to form a vertex region of interest. Averaging across electrodes that show consistent and comparable activity has also been demonstrated to be more reliable than using single electrodes (Huffmeijer et al., 2014). Grand average waveforms were used to select peak latency measurement epochs, see Supplementary Material. P50 was defined as the first positive maximum value following stimulus onset, N1, P2, N2, and P3 peaks were defined as sequential polarity maxima. Peak magnitude was measured as the mean amplitude during epochs defined by 1 SD around the mean peak latency.

To calculate the aMMN, the averaged response to the standard stimuli was subtracted from the deviant stimuli to create a difference waveform. Sequential 1 sample *t*-tests were then applied to the difference waveforms for each group using the method outlined by Guthrie and Buchwald (1991). The consecutive time points necessary to indicate an epoch of significant difference between the standard and deviant responses were obtained from a simulation using an autocorrelation estimated from the data. Intervals with values of *p* < 0.05 that lasted for the required duration, (14 consecutive time points [i.e., 14 ms] for the healthy older adults, 7 for the younger adults), were accepted as significantly different epochs. An aMMN amplitude was then calculated as the mean amplitude of any significant negative deflection in the difference waveform (as identified by the sequential *t*-test procedure) following the N1 peak, and aMMN peak latency as the most negative deflection in the difference wave.

#### 2.3.4 Statistical analysis

For the puretone paradigm, the amplitudes and latencies of the P50, N1, P2, and N2 were examined in a 1-way (age: young vs. old) analysis of variance (ANOVA). For the auditory speech paradigm, the amplitudes and latencies of the 4 major auditory ERPs (P50, N1, P2, and N2) were examined individually in a 2 (age: young vs. old) × 2 (condition: standard vs. deviant) ANOVA. An aMMN and P3a to deviants were examined separately in a 1-way (age: young vs. old) ANOVA.

### 2.4 Results

#### 2.4.1 Puretone ERPs

Averaged ERPs to puretones for younger and older adults are shown in Fig. 1.

There was no effect of age on P50 amplitudes (*F* [1,44] = 0.39, *p* = 0.534); however, older adults showed significantly earlier P50 latencies (*F* [1,44] = 9.04, *p* = 0.004). Older adults displayed a significantly increased N1 amplitude (*F* [1,44] = 7.82, *p* = 0.008), but there were no significant differences in latency (*F* [1,44] = 0.44, *p* = 0.513). There was no effect of age on P2 amplitude (*F* [1,44] = 0.09, *p* = 0.754) or P2 latency (*F* [1,44] = 2.71, *p* = 0.107). There was a strong effect of age on N2 amplitude (*F* [1,44] = 35.38, *p* < 0.001) with no clear N2 peak observable in the older adult group, see Fig. 1. Given the absence of an identifiable N2 peak in older adults, N2 latency differences were not compared.

#### 2.4.2 Auditory speech ERPs

**Fig. 2:**
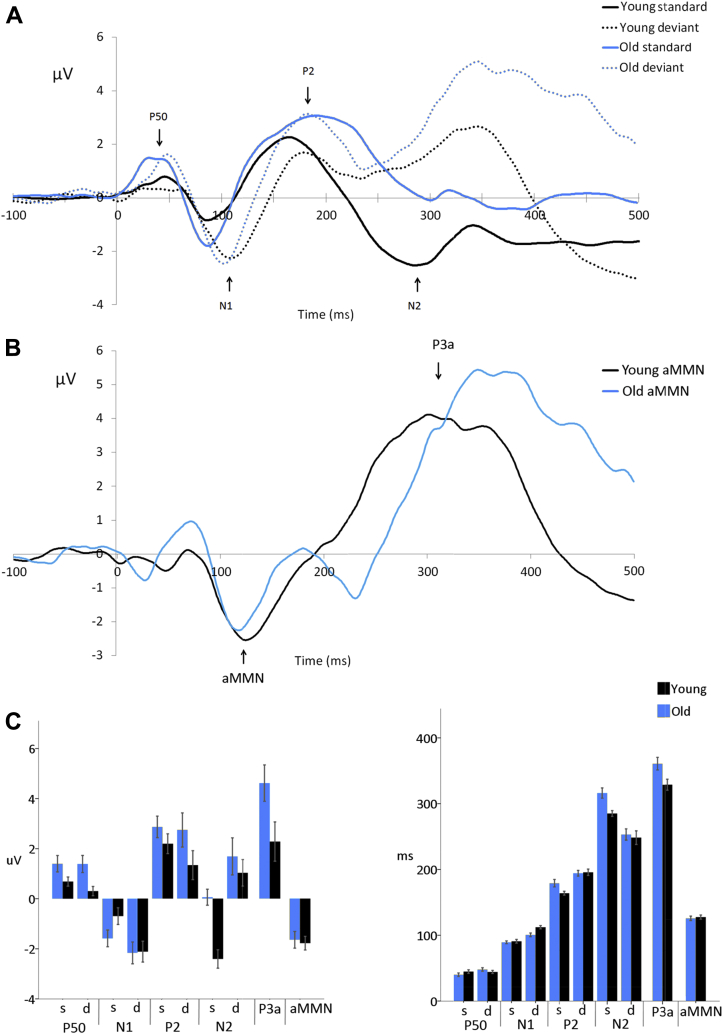
(A) Grand average responses to auditory speech stimuli measured at the vertex region of interest (average of 9 electrodes FC1, FCz, FC2, C1, Cz, C2, CP1, CPz, and CP2) for younger and older adults. (B) Difference waveforms (deviant minus standard) illustrating the aMMN response for younger and older adults. (C) Mean amplitudes and latencies of the ERPs elicited in response to standard (s) and deviant (d) auditory speech stimuli, for younger and older adults. Error bars indicate the standard error of the mean. Abbreviations: aMMN, auditory mismatch negativity; ERPs, event-related potentials.

Averaged ERPs to auditory-only speech for younger and older adults are shown in Fig. 2.

##### 2.4.2.1 P50

There was a significant effect of age and condition on P50 amplitude, that is, older adults showed a significantly increased P50 amplitude compared with younger adults (*F* [1,44] = 4.94, *p* = 0.031), and standard stimuli elicited a significantly increased P50 compared with deviant stimuli (*F* [1,44] = 4.23, *p* = 0.045). There was weak evidence for an interaction between age and condition (*F* [1,44] = 3.74, *p* = 0.060).

There was no significant effect of age (*F* [1,44] = 0.02, *p* = 0.895) or condition (*F* [1,44] = 3.23, *p* = 0.079) on P50 latency. There was a marginally significant interaction between age and condition (*F* [1,44] = 4.31, *p* = 0.044) due to older adults showing a delayed P50 to deviant stimuli compared with younger adults who showed no difference in P50 latency to standard and deviant stimuli.

##### 2.4.2.2 N1

There was no effect of age on N1 amplitude (*F* [1,44] = 0.79, *p* = 0.379). Standard stimuli elicited a significantly reduced N1 compared with deviant stimuli (*F* [1,44] = 35.05, *p* < 0.001). There was a significant interaction between age and condition (*F* [1,44] = 6.24, *p* = 0.016) due to a more pronounced effect of condition on N1 amplitude in younger adults, see Fig. 2.

Older adults showed a significantly earlier N1 (*F* [1,44] = 4.72, *p* = 0.035), standard stimuli elicited an earlier N1 than deviant stimuli (*F* [1,44] = 58.77, *p* < 0.001), and there was a significant interaction between age and condition (*F* [1,44] = 5.58, *p* = 0.023) due to a more pronounced effect of condition on N1 latency in younger adults.

##### 2.4.2.3 P2

There was no significant effect of age (*F* [1,44] = 2.29, *p* = 0.137) or condition (*F* [1,44] = 1.66, *p* = 0.204) on P2 amplitude, and there was no significant interaction between age and condition (*F* [1,44] = 0.96, *p* = 0.332).

There was no significant effect of age on P2 latency (*F* [1,44] = 1.90, *p* = 0.175). Standard stimuli elicited an earlier P2 than deviant stimuli (*F* [1,44] = 25.80, *p* < 0.001). There was no significant interaction between age and condition (*F* [1,44] = 3.07, *p* = 0.087).

##### 2.4.2.4 N2

N2 amplitude was affected by age and condition: older adults showed a significantly reduced N2 amplitude compared with younger adults (*F* [1,44] = 6.79, *p* = 0.012), and standard stimuli elicited an increased N2 compared with deviant stimuli (*F* [1,44] = 28.49, *p* < 0.001). There was weak evidence for an interaction between age and condition (*F* [1,44] = 3.64, *p* = 0.063) with condition having a greater impact on N2 amplitude among younger adults compared with older adults.

Similarly, N2 latency was affected by age and condition: N2 was significantly delayed in older compared with younger adults (*F* [1,44] = 5.48, *p* = 0.024), and for standard compared to deviant stimuli (*F* [1,44] = 32.47, *p* < 0.001). There was no clear evidence for an interaction between age and condition (*F* [1,44] = 2.28, *p* < 0.139).

##### 2.4.2.5 P3a

P3a amplitude was significantly increased (*F* [1,44] = 4.75, *p* = 0.035) and latency significantly delayed in older adults (*F* [1,44] = 5.94, *p* = 0.019).

##### 2.4.2.6 aMMN

Sequential 1 sample *t*-tests identified the mean aMMN duration as 79 ms in younger adults (86–165 ms) and 53 ms (93–146 ms) in older adults. There was no significant effect of age on mean aMMN amplitude (*F* [1,44] = 0.90, *p* = 0.766) or the peak latency of the difference wave (*F* [1,44] = 2.16, *p* = 0.149) during these epochs.

### 2.5 Discussion

Experiment 1 compared ERPs from younger and older adults to puretones, and to simple speech stimuli (syllables /ba/ and /da/) presented in an oddball paradigm. Older adults showed earlier P50 latencies followed by increased N1 amplitudes compared with younger participants for puretones. Similarly, older adults showed increased P50 and N1 amplitudes for auditory speech. Increased amplitudes of early sensory components in older participants under conditions when the HTL was adjusted (auditory speech) or not adjusted for (puretones) demonstrate that the effect was not a simple consequence of physically more intense/louder stimuli (see Supplementary Material for correlational analyses between ERP amplitudes and HTL).

Critically, older adults' N2 response to regular repeating stimuli (i.e., the puretone stimulus and to the standard in the auditory speech paradigm) was absent or strongly reduced compared with younger adults. As expected, no N2 peak was found in response to deviant stimulus in either group (Bertoli and Probst, 2005). Older adults' P3a responses to deviant stimuli were increased and delayed. The combination of increased early sensory responses P50 and N1, absent or strongly reduced N2 to standard stimuli, and an increased P3a to deviant stimuli in older participants points to a decreased ability to inhibit responses to regular repeating information, and greater attentional capture from rare task-irrelevant information. There was no effect of age on P2 amplitudes or latencies in either paradigm, and aMMN in older adults was equivalent in amplitude and peak latency to that in younger adults. The implications of these findings for cognitive theories of aging are discussed in full in the Section 4

## 3 Experiment 2

Experiment 2 extended experiment 1 into the audiovisual domain, using the same participants as in experiment 1. In experiment 2, we manipulated the congruency of the visual information accompanying the auditory speech stimulus to examine the influence of “relevant” versus “distracting” visual information. We expected that facilitatory effects of congruent visual information will be preserved in older adults, however that the interference from incongruent visual information will increase with age.

### 3.1 Stimuli

#### 3.1.1 Audiovisual speech

**Fig. 3:**
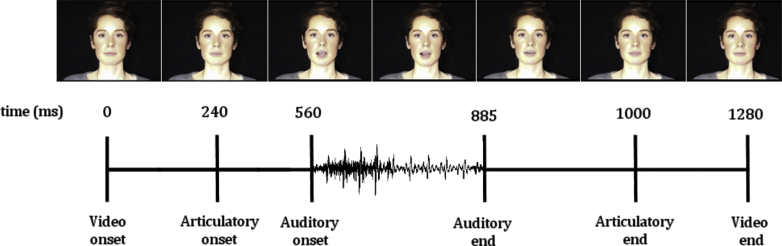
Video timings for standard and deviant stimuli.

Stimuli were digitally recorded videos (frame rate: 25 images/s; audio sample rate: 44.1 KHz in 16 bits) of a female speaker pronouncing the syllables /ba/ and /ga/. Videos were digitally edited using Pinnacle software v.15 (Corel Inc) to ensure that the onset of syllabic articulatory movements, auditory onset, and auditory duration in both videos were identical. The videos were 1280 ms long with articulatory onset at 240 ms and auditory onset at 560 ms, see Fig. 3. The duration of the auditory stimuli was 325 ms.

The standard stimulus was the video of the speaker pronouncing /ba/. The deviant stimulus was created by overdubbing the audio track from the /ba/ video onto the silent video of the speaker pronouncing /ga/. The combination of auditory /ba/ and visual /ga/ typically elicits the McGurk illusion (McGurk and MacDonald, 1976) fused percept of /da/. A summary of the syllables and percepts in both auditory and audiovisual paradigms is presented in Table 1. The mean ISI between videos of 620 ms varied randomly between 520 and 720 ms. During the ISI, a still frame of the speaker's face was presented on screen. This image was matched to the first and last frame of the videos, creating the impression of continuous natural speech, that is, no visual onset or offset. Stimuli were matched for auditory intensity using Praat software (Boersma and Weenink, 2009).

### 3.2 Procedure

#### 3.2.1 Behavioral discrimination task

Participants completed a discrimination task at the end of the EEG recording session. They were presented 25 congruent /ba/ and 50 incongruent McGurk /da/ videos, identical to those used in the audiovisual paradigm. In addition, 25 congruent /ga/ videos were presented (i.e., /ga/ video with congruent /ga/ auditory stimulus). Participants were instructed to watch the speaker's face at all times and to report the syllable they heard using a handheld response button box. The videos were presented in a fully randomized sequence lasting approximately 3 minutes.

#### 3.2.2 Audiovisual speech

The videos were presented on a computer monitor 0.5 m directly in front of the participant. The auditory stimuli were presented binaurally through headphones at approximately 60-dB SPL above the participant's HTL following the same adjustment procedure as in experiment 1. Participants were instructed to attend to the speaker, listen to what was said and watch the speaker's face at all times. The standard /ba/ and deviant /da/ (i.e., McGurk) stimuli were presented in a pseudo-random sequence with at least 2 standards preceding each deviant. The ratio of standards:deviants was 8:1. Eight hundred and ninety six standards and 112 deviants were presented in 2 blocks lasting 12 minutes each. EEG recording techniques and analyses were identical to experiment 1. Epochs from −100 ms to 1500 ms were used for the audiovisual data in experiment 2.

#### 3.2.3 The influence of visual information on speech processing in aging

To examine the role of visual information on speech processing in aging, we compared responses to the standard stimuli across the auditory and audiovisual paradigms as they were perceptually and acoustically identical in both paradigms, that is, the participant heard and perceived a /ba/ syllable. Deviant stimuli were not compared as although they were perceptually the same (i.e., auditory = “spoken” /da/, audiovisual = “illusory” /da/), they were acoustically different (i.e., auditory deviant = “spoken” /da/, audiovisual deviant = “spoken” /ba/).

### 3.3 Statistical analysis

The amplitudes and latencies of the 4 major auditory ERPs (P50, N1, P2, and N2) were examined individually in a 2 (age: young vs. old) × 2 (condition: standard vs. deviant) ANOVA. The influence of visual information on auditory processing and its interaction with age was examined using a mixed design ANOVA. A 2 × 2 ANOVA with factors group (young/old) and visual information (absent [=auditory]/present [=audiovisual]) was performed for the P50, N1, P2, and N2 responses to standard stimuli. No MMN or P3a response was observed; however, an extended period of positivity following the P2 peak was observed in the older adults' responses. To quantify group differences in this response, the mean amplitude of the difference wave (deviant minus standard) between 800–1500 ms was examined in 1-way (age: young vs. old) ANOVA.

### 3.4 Results

#### 3.4.1 Behavioral discrimination task

One older adult did not complete the task due to tiredness. There was no significant difference between the groups in the number of McGurk /da/ illusions perceived (Younger mean (M) = 72% SE = 8.78, Older M = 75% SE = 6.12; *t* [1,43] = −0.33, *p* = 0.740), or the number of congruent /ba/ or /ga/ syllables correctly identified (Younger M = 99% SE = 0.3, Older M = 95% SE = 2.5; *t* [1,43] = 1.48, *p* = 0.146).

#### 3.4.2 Audiovisual speech

##### 3.4.2.1 P50

Older adults showed significantly larger P50 amplitudes compared with younger adults (*F* [1,44] = 4.71, *p* = 0.035). Standard stimuli elicited a significantly reduced P50 compared with deviant stimuli, (*F* [1,44] = 9.82, *p* = 0.003), there was no significant interaction between age and condition (*F* [1,44] = 0.59, *p* = 0.447).

There was weak evidence for an effect of age on P50 latency with older adults showing delayed P50 responses (*F* [1,44] = 3.59, *p* = 0.065). Standard stimuli elicited an earlier P50 than deviant stimuli (*F* [1,44] = 29.93, *p* < 0.001), there was no significant interaction between age and condition (*F* [1,44] = 0.33, *p* = 0.571).

##### 3.4.2.2 N1

Older adults showed a significantly increased N1 amplitude compared with younger adults (*F* [1,44] = 5.24, *p* = 0.027), standard stimuli elicited a significantly increased N1 amplitude compared with deviant stimuli (*F* [1,44] = 44.27, *p* < 0.001). There was no significant interaction between age and condition (*F* [1,44] = 0.36, *p* = 0.553).

There was no significant effect of age on N1 latency (*F* [1,44] = 0.45, *p* = 0.505), standard stimuli elicited a significantly earlier N1 than deviant stimuli (*F* [1,44] = 46.90, *p* < 0.001). There was no significant interaction between age and condition (*F* [1,44] = 0.42, *p* = 0.521).

##### 3.4.2.3 P2

There was no significant effect of age (*F* [1,44] = 1.57, *p* = 0.216) on P2 amplitude. Standard stimuli elicited a reduced P2 compared with deviant stimuli (*F* [1,44] = 7.99, *p* = 0.007). There was no significant interaction between age and condition (*F* [1,44] = 0.06, *p* = 0.811).

Older adults showed a significantly delayed P2 latency (*F* [1,44] = 16.65, *p* < 0.001). Standard stimuli elicited significantly earlier P2 than deviant stimuli (*F* [1,44] = 41.54, *p* < 0.001). There was no significant interaction between age and condition (*F* [1,44] = 2.49, *p* = 0.122).

##### 3.4.2.4 N2

**Fig. 4:**
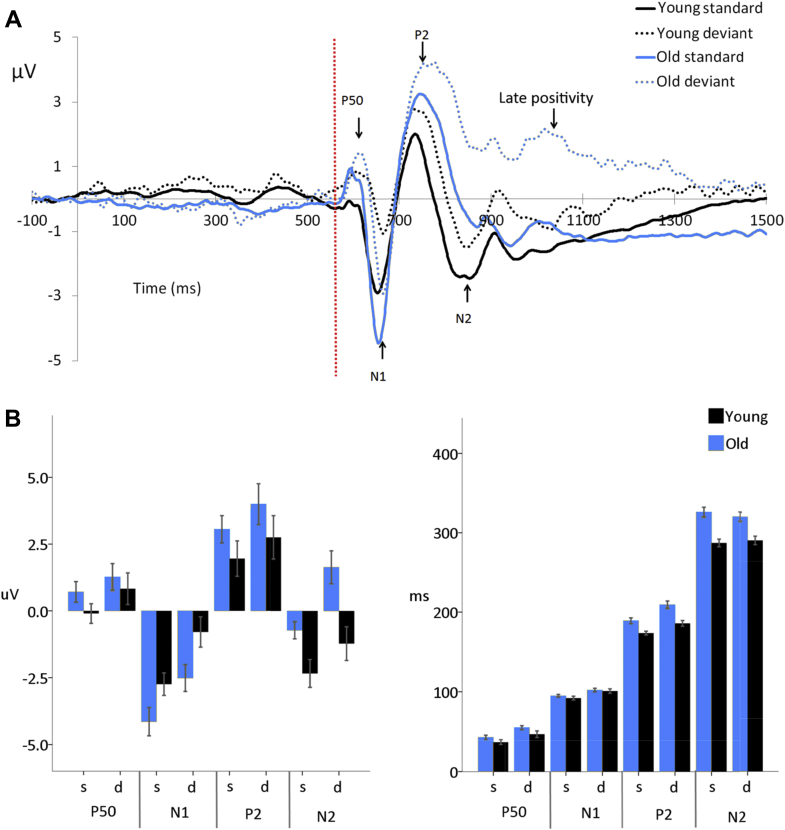
(A) Grand average responses to audiovisual speech stimuli measured at the vertex region of interest (average of 9 electrodes FC1, FCz, FC2, C1, Cz, C2, CP1, CPz, and CP2) for younger and older adults. The dotted red line indicates auditory onset. (B) Mean amplitudes and latencies of the ERPs elicited in response to standard (s) and deviant (d) audiovisual speech stimuli, for younger and older adults. Error bars indicate the standard error of the mean. Abbreviation: ERPs, event-related potentials. (For interpretation of the references to color in this figure legend, the reader is referred to the Web version of this article.)

Older adults showed a significantly reduced N2 amplitude compared with younger adults (*F* [1,44] = 11.24, *p* = 0.002), and standard stimuli elicited an increased N2 compared with deviant stimuli (*F* [1,44] = 24.13, *p* < 0.001). There was weak evidence for an interaction between age and condition (*F* [1,44] = 3.07, *p* = 0.087), with older adults showing a greater impact of condition on N2 amplitude compared with younger adults who showed little difference, see Fig. 4.

Older adults showed a significantly delayed N2 (*F* [1,44] = 25.01, *p* < 0.001). There was no effect of condition (*F* [1,44] = 0.06, *p* = 0.815) and no interaction between age and condition (*F* (1,44) = 1.03, *p* = 0.316).

##### 3.4.2.5 aMMN, P3a, and late positivity

No MMN or P3a response was observable in either the younger or older adults' data. There was a prolonged late positivity in response to the deviant stimuli, beginning at the P2 peak and lasting for the remainder of the epoch that was significantly larger among older adults (*F* [1,44] = 7.32, *p* = 0.010).

#### 3.4.3 The influence of visual information on speech processing in aging–comparison of auditory versus audiovisual ERPs

To examine the role of visual information on speech processing in aging, we compared responses to the standard stimuli across the auditory (experiment 1) and audiovisual (experiment 2) paradigms. Recall that standard stimuli in the auditory and audiovisual paradigms were perceptually and acoustically identical, that is, the participant heard and perceived a /ba/ syllable. Deviant stimuli were not compared across the auditory (experiment 1) and audiovisual (experiment 2) paradigms, as the stimuli were acoustically different, that is /da/ in experiment 1 and /ba/ in experiment 2.

##### 3.4.3.1 P50

**Fig. 5:**
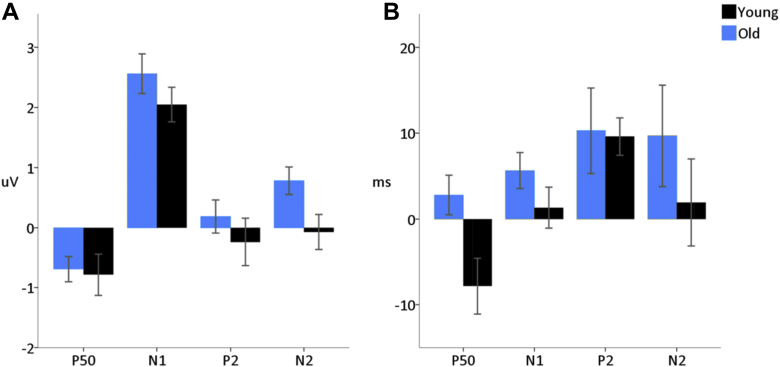
(A) Mean amplitude differences and (B) latency differences (audiovisual–auditory) for younger and older adults' responses to standard stimuli in the auditory versus audiovisual paradigms measured at the vertex region of interest (average of 9 electrodes: FC1, FCz, FC2, C1, Cz, C2, CP1, CPz, and CP2). For all components, a positive value indicates an increased amplitude or delayed latency of the peak in the presence of visual information; a negative value indicates decreased amplitude or an earlier peak latency. Error bars indicate standard error of the mean.

P50 amplitude was significantly reduced in the presence of visual information (*F* [1,44] = 14.44, *p* = 0.001), there was no significant effect of age (*F* [1,44] = 2.98, *p* = 0.091), and there was no significant interaction between age and visual information (*F* [1,44] = 0.06, *p* = 0.812), see Fig. 5.

There was no main effect of visual information (F [1,44] = 1.67, *p* = 0.203) or age (F [1,44] = 0.040, *p* = 0.843) on P50 latency. However, there was a significant interaction between age and visual information with younger adults showing an earlier P50 when visual information was present (F [1,44] = 7.47, *p* = 0.009), see Fig. 5.

##### 3.4.3.2 N1

The presence of visual information increased N1 amplitude significantly (*F* [1,44] = 103.9, *p* < 0.001). Older adults showed larger N1 amplitudes across paradigms (*F* [1,44] = 4.16, *p* = 0.047), and there was no significant interaction between age and visual information, (*F* [1,44] = 1.29, *p* = 0.262).

The presence of visual information significantly delayed N1 latencies (*F* [1,44] = 4.85, *p* = 0.033). There was no significant effect of age (*F* [1,44] = 0.49, *p* = 0.826) or interaction between age and visual information (*F* [1,44] = 1.85, *p* = 0.181).

##### 3.4.3.3 P2

There was no significant effect of visual information on P2 amplitude (*F* [1,44] = 0.01, *p* = 0.915), no effect of age (*F* [1,44] = 1.69, *p* = 0.200), and no significant interaction between age and visual information (*F* (1,44) = 0.82, *p* = 0.369).

The presence of visual information resulted in significantly delayed P2 latencies (*F* [1,44] = 10.94, *p* = 0.002), and older adults showed significantly delayed P2 latencies (*F* [1,44] = 8.91, *p* = 0.005) across paradigms. There was no significant interaction between age and visual information, (*F* [1,44] = 0.01, *p* = 0.911).

##### 3.4.3.4 N2

There was some evidence for the presence of visual information to increase N2 amplitude, although the effect was marginally significant (*F* [1,44] = 3.79, *p* = 0.058). Older adults showed significantly reduced N2 amplitudes across paradigms (*F* [1,44] = 16.26, *p* < 0.001). There was a significant interaction between age and the presence of visual information (*F* [1,44] = 5.45, *p* = 0.024) as visual information increased N2 amplitude in older but not younger adults.

The presence of visual information did not have a significant effect on N2 latencies (*F* [1,44] = 2.07, *p* = 0.157). Older adults showed significantly delayed N2 latencies across paradigms (*F* [1,44] = 18.85, *p* < 0.001), although given the absence of a clear N2 peak latency measures are less reliable. The interaction between age and visual information was not significant (*F* [1,44] = 0.92, *p* = 0.342).

### 3.5 Discussion

In experiment 2, older adults showed equivalent behavioral sensitivity to the McGurk illusion as younger adults. In terms of ERPs, experiment 2 replicated the pattern of inhibitory deficit in older adults observed in experiment 1. Older adults showed significantly increased P50 and N1 amplitudes compared with younger adults and no observable N2 peak to standard stimuli. The effect of congruency of visual information was also considerable, with congruent visual information increasing N1 amplitudes in both younger and older adults compared with incongruent visual information.

Examination of the effects of the presence of visual information (via comparison of ERPs in response to standard stimuli in the audiovisual paradigm in experiment 2 vs. auditory paradigm in experiment 1) demonstrates similarly enhanced N1 amplitudes when (congruent) visual information is added to accompany an auditory stimulus in younger and older adults. This suggests that the facilitatory effect of visual information is maintained in aging.

## 4 General discussion

Across puretone, auditory and audiovisual speech paradigms older adults showed a consistent pattern of inhibitory deficits, manifested as increased P50 and/or N1 amplitudes and an absent or significantly reduced N2. Experiment 1 demonstrated that this pattern was present in auditory processing regardless of adjustment for HTL or the acoustic complexity of the stimuli, whereas experiment 2 demonstrated that this pattern extended to audiovisual processing. We propose that these findings provide evidence for IDH, in particular for the claim that older adults are less able to regulate the access to attentional focus of afferent sensory information.

Experiment 2 also provided further insights into the role of visual information in auditory processing in aging. Congruent articulatory visual information enhanced N1 amplitudes for the audiovisual compared with auditory speech in both younger and older adults. When the effect of congruent and incongruent visual information were compared in audiovisual processing, incongruent information resulted in a prolonged, late positivity in older adults.

An increase in early auditory ERP amplitudes in healthy older adults as compared with younger adults has been previously demonstrated (see Ceponiene et al., 2008, Friedman, 2008) and proposed to reflect a lack of inhibitory regulation of afferent sensory information by the prefrontal cortex. The absence of the standard N2 in older adults is less well documented and often overlooked in analyses in favor of examining responses to deviants or targets (e.g., Daffner et al., 2015). When addressed directly, the absence or reduction of the N2 has been associated with poorer gap detection and processing speed (Harris et al., 2012) and proposed to reflect a lack of frontal inhibition among older adults (Bertoli and Probst, 2005, Ceponiene et al., 2008, Getzmann et al., 2015, Zendel and Alain, 2014). The N350 is a possible equivalent observed in sleep EEG, it is observed during sleep and sleepiness and reflects a mechanism contrary to attention, preventing conscious processing of stimuli and facilitating falling asleep (Kállai et al., 2003). It is speculative and beyond the remit of the present study to link the inhibitory negative components observed in sleep with the N2 observed in younger adults, but is a deserved avenue for future research. We propose that the standard N2 in our present study represents a neural “stop-signal” that serves to prevent further unnecessary processing of a repetitive stimulus and that it is consistently and markedly absent in older adults across a variety of auditory processing tasks. This is a critical element of the first aspect of inhibition, that is, controlling access of irrelevant information to the focus of attention and working memory (Guerreiro et al., 2010, Hasher et al., 2007) and is an automatic and integral part of sensory processing.

Note that in experiment 2, younger adults showed a clear N2 to the deviant stimulus, a finding that we did not predict. The incongruity of the audio-visual deviant affected even the earliest ERPs thus making effects in later windows such as the N2 window difficult to interpret (see discussion of the absent audiovisual mismatch negativity below). One possibility that is suggested by the presence of an N2 to both standards and deviants is that N2 is based on the basis of the auditory stimulus only (/ba/, i.e., identical for the standard and deviant), that is, it remains relatively unaffected by audiovisual binding compared to earlier (P50 and N1) components. Another possibility is that the N2 to deviants is an N2b, reflecting the direct attention to stimuli (Patel and Azzam, 2005). This is notably different from the auditory speech paradigm, in which no N2 was observed to deviant stimuli, raising the possibility that attentional focus was greater to the audiovisual speech paradigm.

Experiment 2 provides support for previous assertions that older adults maintain the ability to process relevant information yet are more susceptible to the distracting and interfering effects of irrelevant information (e.g., Cashdollar et al., 2013, Gazzaley et al., 2005, Gazzaley et al., 2008). Congruent articulatory visual information significantly increased N1 amplitude in both younger and older adults compared with auditory processing alone, demonstrating that older adults are still able to facilitate and enhance the processing of relevant (visual) information. This adds to the behavioral findings of Sommers et al. (2005) in which older adults received an equivalent visual “enhancement” of auditory processing in noise to younger adults. It should be noted that the interpretation of this increased N1 as a facilitatory effect, rather than as a marker of inhibitory deficit, is due to the underlying assumption that the younger adults' ERPs are the default “healthy” response, that is, because younger adults show an increase in N1 amplitude in the audiovisual paradigm, this is the baseline against which to compare.

Older adults’ P50 and N1 latencies were delayed, and younger adults showed earlier P50 latencies, in the presence of visual information, suggesting there may be a temporal cost to maintaining the benefit of congruent visual information with age. This adds to the findings of Diederich et al. (2008) who demonstrated that compared with younger adults, older adults showed slower overall multisensory integration during a saccadic reaction time task yet showed a greater neural benefit from congruent compared with incongruent multisensory stimuli.

Older adults also perceived the McGurk illusion with comparable frequency to younger adults, replicating previous behavioral findings demonstrating maintained audio-visual integration in healthy aging (Cienkowski and Carney, 2002, Huyse et al., 2014). The increase in N1 amplitude as a consequence of the presence of congruent visual articulatory information is contrary to some previous ERP studies of audio-visual processing (e.g., van Wassenhove et al., 2007, Winneke and Phillips, 2011) in which audio-visual N1 amplitudes were reduced and latencies shortened compared with those in the auditory-alone condition, a pattern interpreted as reflecting increased neural efficiency. However, a critical difference between the present study and previous studies is that participants were not asked to respond to stimuli, and there was no distinct visual onset, that is, the speaker's face was onscreen at all times. Therefore, the interaction of attention, task demands, and the alerting effect of a distinct visual onset may affect the influence of predictive visual information on the timing and magnitude of auditory ERPs (Kok et al., 2012).

Among older adults only, incongruent visual information resulted in a prolonged late positive deflection following the P2 that lasted the duration of the epoch. We suggest that the late positive deflection observed in the present study may reflect the increased processing effort required by older adults to reanalyse/revise mismatching visual articulatory and auditory information. Such increased processing has been previously demonstrated in psychophysical tasks in which older adults show a larger impact of distracting information on perceptual abilities as a result of prolonged processing of distractors (Cashdollar et al., 2013). Most relevantly, a similar late positivity has been demonstrated by Liu et al (2011) in response to incongruent audio-visual scenarios in which the action in the video (e.g., fireworks explode) mismatched the preceding audio (e.g., shattering glass). The authors related their finding to the linguistic “P600” effect, which is known to reflect a reanalysis or revision of incongruent syntactic information into a plausible or meaningful arrangement (Osterhout and Holcomb, 1992). Further research is needed to elucidate whether the late positivity observed in our study belongs to the same family of effects.

Older adults showed an equivalent aMMN to younger adults, contrary to many previous studies of aMMN (e.g., Kiang et al., 2009), and contrary to our predictions. Interestingly, the MMN response appears to be robust to the preceding impact of inhibitory deficit on N1 amplitudes. The adjustment of HTL may explain the discrepancy with previous findings. It is well-known that aMMN amplitudes increase as the standard and deviant become more discriminable (Schröger et al., 1992). Therefore, it is possible that previous studies that did not adjust for individual HTLs and simply ensured that all participants had HTLs below a common threshold (e.g., Alain et al., 2004, Cooper et al., 2006, Kiang et al., 2009) were presenting less discriminable stimuli for the older adults, resulting in a reduced aMMN. The use of natural speech stimuli, rather than puretones, may have also contributed to the maintenance of aMMN in older adults in the present study. The few electrophysiological studies on speech MMN in older adults show conflicting results, e.g., Bellis et al. (2000) found no effects of age on MMN to syllables, whereas Cheng et al. (Cheng et al., 2015) showed a reduction amongst older adults in the magnetic MMN to speech syllables. In addition the measurement of the aMMN differed from previous studies, that is, we used sequential *t*-tests to identify the duration of the aMMN response and then measured the mean amplitude during this bespoke epoch as opposed to taking mean amplitudes during arbitrarily defined epochs, e.g., from 100-200 ms, or for 100 ms following the N1 peak. In fact, if such arbitrarily defined fixed epochs were used to measure the aMMN in the present study it would show a lower mean aMMN amplitude in older adults. This is because the older adults' aMMN response was 26 ms shorter than that in younger adults, which would have resulted in a lower mean aMMN amplitude for older than younger adults if it were measured using a fixed window. By accurately identifying the duration of the aMMN response we are more able to accurately assess its magnitude in each group. A possible interpretation of the current data is that aMMN is impacted by healthy aging, but it is the duration rather than the amplitude of the response that is reduced. For this hypothesis to be tested further, aMMN paradigms should be optimized to enable calculation of the duration of individuals' aMMN responses as well as group responses.

Interestingly, despite the lack of large differences in the MMN, older adults also showed increased and delayed P3a responses to deviant stimuli, suggesting greater neural resources devoted to the attentional orientation toward deviant stimuli. These findings complement previous studies showing increases in the P3a in older adults (e.g., Alperin et al., 2014, Daffner et al., 2015) and provide further support for the idea that older adults find it harder to ignore task-irrelevant information; a fundamental element of the IDH.

No MMN response was observed in the audiovisual paradigm. In the present study, congruency of visual information significantly affected both the P50 and N1 peaks, consequently the pre MMN epoch (i.e., from stimulus onset to the N1 peak) was not equal for standard and for deviants. If deviant stimuli did elicit an MMN response it may have been masked by the preceding peak amplitude differences. Previous studies that have demonstrated a McGurk illusion MMN (Colin et al., 2002, Kislyuk et al., 2008, Saint-Amour et al., 2007) had 2 important methodological differences. First, there was a distinct visual onset and offset of the visual information, that is, the speaker's image appeared at the start and disappeared at the end of each trial. Second, visual-only ERPs were subtracted from the audio-visual ERPs to calculate the auditory ERPs. In the present study, the speaker's face remained onscreen at all times, so there was no distinct visual onset and offset. This provided more ecologically valid speech stimuli, avoided the confounding effect of visual-onset ERPs but may have compromised the measurement of the audiovisual MMN.

There are some limitations to the present study. First, attention was not directly controlled for. This raises the possibility that increased early sensory P50 and N1 amplitudes may have been a consequence of additional attentional effort among the older adult group, for example, as an attempt to compensate for any deterioration in hearing ability. Note that the absence/reduction of the standard N2 in the older participants cannot be solely an outcome of extra attention because Bertoli and Probst (2005) previously demonstrated such an age-related N2 reduction in both attended and unattended auditory oddball paradigms. Hence, the age-related reduction in the standard N2 cannot be explained away by extra attention from the older participants but rather a genuine difference in the “stop-signal” process that the standard N2 reflects. Second, there were no behavioral measures of performance to assess the consequences of any inhibitory deficit in early sensory processing. Third, the ISIs were not equal in the auditory speech and audiovisual speech paradigms, possibly introducing a confound in the comparison of ERP amplitudes across paradigms. Future studies should investigate the role of attention and ISIs on inhibition and facilitation in older adults and explicitly examine the link between neural and behavioral responses. Finally, to further characterize oddball responses in both audio-visual and auditory-only paradigms, an additional audiovisual condition with a congruent deviant stimulus, e.g., visual /da/ + auditory /da/, would allow for the comparison of both standard and deviant stimuli responses across paradigms.

In summary, we have demonstrated a pattern of age-related auditory processing that is consistent with the IDH. Older adults consistently show increased early sensory ERPs, and an absence of a standard N2 which in combination reflects a deficit in the frontal regulation of sensory processing. Older adults are still able to use congruent visual articulatory information to aid auditory processing, but at a temporal cost, and appear to require greater neural effort to resolve conflicts generated by incongruent visual information. Future work should focus on establishing the neural mechanisms of frontal regulation of sensory processing, and how these mechanisms change with age.

## Disclosure statement

The authors have no conflicts of interest to disclose.

## Figures and Tables

**Table 1:** Visual, auditory, and perceptual characteristics of the standard and deviant stimuli for the audiovisual speech

Condition	Auditory	Visual	Percept
Standard	/ba/	/ba/	/ba/
Deviant	/ba/	/ga/	/da/
