# Supplementary material for: Auditory perception in the aging brain: the role of inhibition and facilitation in early processing
Source: Neurobiol Aging. 2016 Nov;47:23–34. doi: 10.1016/j.neurobiolaging.2016.06.022 (PMC5082767; doi:10.1016/j.neurobiolaging.2016.06.022)
Supplement: Supplementary Material [file mmc1.docx]

Supplementary Information – ERP measurement epochs

| **Peak latency**  **measurement epoch (ms)** | **Puretone**  **(Experiment 1)** | **Auditory**  **(Experiment 1)** | **Audiovisual**  **(Experiment 2)** |
| --- | --- | --- | --- |
| **P50** | 20-75 | 20-65 | 8-79 |
| **N1** | 76-115 | 60-150 | 54-132 |
| **P2** | 116-170 | 115-235 | 123-291 |
| **N2** | 171-260 | 180-350 | 179-434 |
| **P3a** | na | 238-550 | na |
| **aMMN** | na | 86-165 | na |
| Table 1: Measurement epochs (ms) for peak latency of ERPs following inspection of grand average waveforms. | | | |

| **Peak amplitude**  **measurement epoch (ms)** | | **Puretone**  **(Experiment 1)** | | **Auditory**  **(Experiment 1)** | | **Audiovisual**  **(Experiment 2)** | |
| --- | --- | --- | --- | --- | --- | --- | --- |
|  |  | **Young** | **Old** | **Young** | **Old** | **Young** | **Old** |
| **P50** | **Standard** | 47-63 | 36-56 | 33-57 | 27-53 | 584-610 | 590-616 |
|  | **Deviant** | na | na | 33-55 | 35-61 | 589-625 | 602-628 |
| **N1** | **Standard** | 87-104 | 85-103 | 78-104 | 78-100 | 642-662 | 646-664 |
|  | **Deviant** | na | na | 101-123 | 87-115 | 649-673 | 650-674 |
| **P2** | **Standard** | 138-156 | 140-164 | 153-177 | 150-208 | 723-745 | 731-768 |
|  | **Deviant** | na | na | 174-218 | 173-217 | 730-762 | 747-793 |
| **N2** | **Standard** | 198-228 | 201-257 | 265-305 | 276-356 | 825-869 | 855-917 |
| **P3a** | **Deviant** | na | na | 292-366 | 312-410 | na | na |
| Table 2: Measurement epochs (ms) for peak mean amplitude of ERPs. Range based on one standard deviation around the mean peak latency. | | | | | | | |

Supplementary information – ERP Hearing threshold correlational analyses


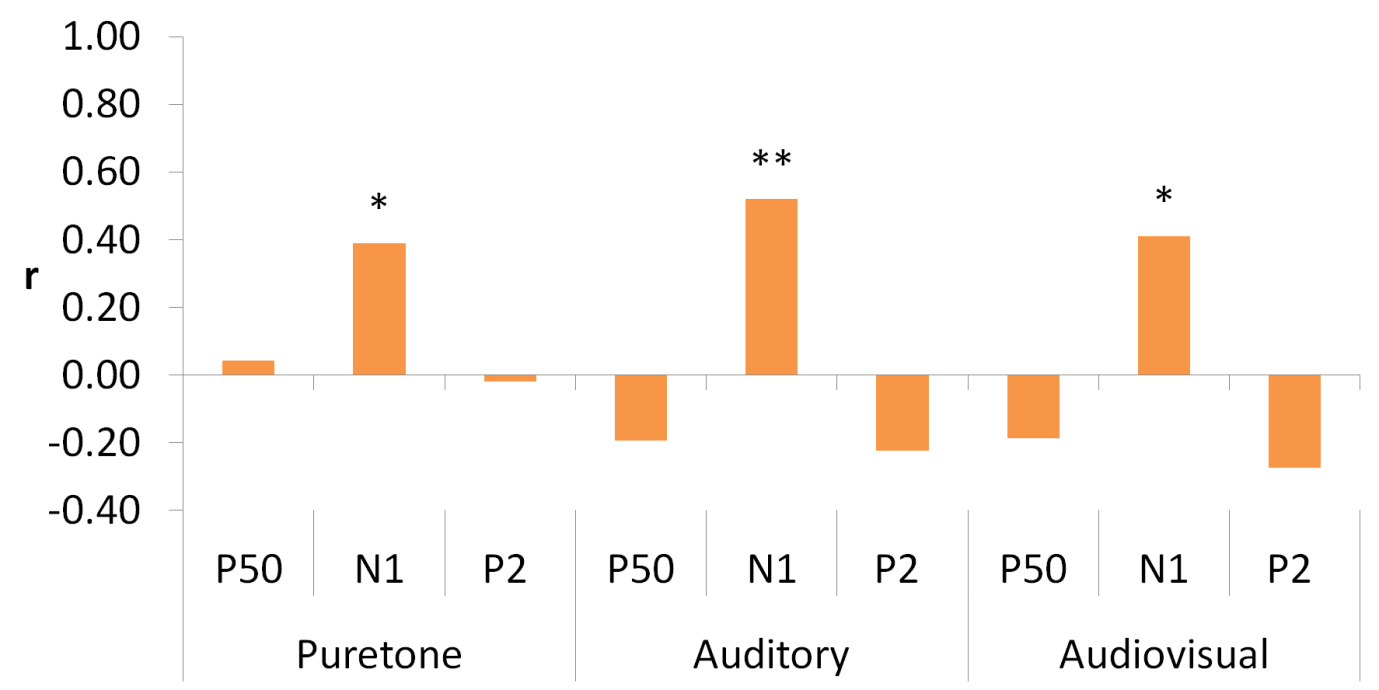


**Figure 1.** Pearson’s r correlations between mean decibel increase in stimulus intensity for the auditory and audiovisual stimuli and peak amplitude measures for responses to standard stimuli in older adults. Note that values for negative peaks have been reversed so that a positive correlation equates to an enhanced peak amplitude irrespective of polarity across all components. * *p* < 0.05 ** *p* < 0.01
